# IL-4Rα-Associated Antigen Processing by B Cells Promotes Immunity in *Nippostrongylus brasiliensis* Infection

**DOI:** 10.1371/journal.ppat.1003662

**Published:** 2013-10-24

**Authors:** William G. C. Horsnell, Matthew G. Darby, Jennifer C. Hoving, Natalie Nieuwenhuizen, Henry J. McSorley, Hlumani Ndlovu, Saeeda Bobat, Matti Kimberg, Frank Kirstein, Anthony J. Cutler, Benjamin DeWals, Adam F. Cunningham, Frank Brombacher

**Affiliations:** 1 International Centre for Genetic Engineering and Biotechnology (ICGEB) and Institute of Infectious Disease and Molecular Medicine (IIDMM), Division of Immunology, University of Cape Town, Cape Town, South Africa; 2 Institute of Immunology and Infection Research, University of Edinburgh, Ashworth Laboratories, Edinburgh, United Kingdom; 3 School of Immunity and Infection and Medical Research Council Centre for Immune Regulation, University of Birmingham, Birmingham, United Kingdom; University of Medicine and Denistry New Jersey, United States of America

## Abstract

In this study, B cell function in protective T_H_2 immunity against *N. brasiliensis* infection was investigated. Protection against secondary infection depended on IL-4Rα and IL-13; but not IL-4. Protection did not associate with parasite specific antibody responses. Re-infection of B cell-specific IL-4Rα^−/−^ mice resulted in increased worm burdens compared to control mice, despite their equivalent capacity to control primary infection. Impaired protection correlated with reduced lymphocyte IL-13 production and B cell MHC class II and CD86 surface expression. Adoptive transfer of in vivo *N. brasiliensis* primed IL-4Rα expressing B cells into naïve BALB/c mice, but not IL-4Rα or IL-13 deficient B cells, conferred protection against primary *N. brasiliensis* infection. This protection required MHC class II compatibility on B cells suggesting cognate interactions by B cells with CD4^+^ T cells were important to co-ordinate immunity. Furthermore, the rapid nature of these protective effects by B cells suggested non-BCR mediated mechanisms, such as via Toll Like Receptors, was involved, and this was supported by transfer experiments using antigen pulsed Myd88^−/−^ B cells. These data suggest TLR dependent antigen processing by IL-4Rα-responsive B cells producing IL-13 contribute significantly to CD4^+^ T cell-mediated protective immunity against *N. brasiliensis* infection.

## Author Summary

Parasitic nematode infections are an extremely important global public health problem. Infections by hookworms and roundworms for example cause anemia, widespread developmental problems and devalued immunity against bacterial infections such as salmonella and tuberculosis. Although treatable with drugs, parasitic nematode re-infections occur as humans do not develop protective immunity. Ultimately, the public health burden caused by these infections will be best controlled by the development of vaccines against nematode infections. For these to be effective, it is important to understand how the various components of the immune system can respond to infection. In this study, we show that B cells, which typically protect against infection by producing antibodies, can also protect against an experimental hookworm like nematode infection by additional mechanisms. This form of protection instead depended on B cells producing cytokines associated with parasitic nematode expulsion and also by providing T cells with specific instruction. Together, these B cell driven responses lead to a rapid resolution of the infection. These important findings indicate that vaccination strategies against nematode parasites such as hookworms need to understand immune responses other than antibody to be optimally protective.

## Introduction

Parasitic nematode infections are a significant global public health burden. Infections with *Ascaris lumbricoides*, *Trichuris trichiura* and the hookworms *Ancylostoma duodenale* and *Necator americanus* occur in a third of the world's population [1]. Individuals frequently suffer from repeated infections and do not develop robust immunity against re-infection [2]. Such infections are significant causes of morbidity, with hookworm infections, for example, being a major cause of childhood anemia in many endemic areas [3]. Effects on cognitive development, as a result of repeated childhood infections have been reported [4], and parasitic larval migrations through the host may exacerbate chronic lung pathologies in endemic areas [5], [6]. To date no licensed vaccines exist against these parasites. To accelerate their development a detailed understanding of host immunity is essential, especially extra intestinal immunity against infective stage larvae [7]. Studies in humans and experimental models of infection have established that T_H_2 immune responses drive host resolution of primary infections [8], [9].

Key to effective expulsion of murine model parasites, such as *Nippostrongylus brasiliensis*, *Heligmosomoides polygyrus* and *Trichuris muris*, is host expression of IL-4Rα [10]. IL-4Rα is an essential component of the heterodimeric receptors required for IL-4 and IL-13 signalling, which ultimately drive host immune polarisation to T_H_2. Use of IL-4Rα^−/−^ mice has clearly demonstrated an absolute requirement for IL-4Rα expression in resolving primary nematode infections. This is dependent on IL-4Rα expression on non-hematopoietic cells [11] including smooth muscle cells [12] and epithelial cells [13], [14]. However, IL-4Rα expression on hematopoietic cells does impact on the magnitude of the hosts T_H_2 response to *N. brasiliensis*. For example, disruption of IL-4Rα expression on CD4^+^ T-cells results in a significantly reduced T_H_2 response to primary *N. brasiliensis* infection [15] and contributes to optimal control of secondary infection [16]. However, it is not known how IL-4Rα expression on other hematopoietic cells contributes to protection from *N. brasiliensis* re-infection.

Our understanding of cellular mechanisms underlying protective immunity to helminth re-infection has, until recently, been limited. Protective immunity to nematode infection can occur both in the intestine, in the case of primary *N. brasiliensis* infection and both primary and secondary *H. polygyrus* infections, while immunity to secondary *N. brasiliensis* infections occurs in the lung. In the case of the strictly intestinal parasitic nematode *H. polygyrus*, rapid resolution of re-infection requires alternatively activated macrophages [17], CD4^+^ T cells [18], parasite specific type 2 antibody responses [19], [20] and B cell cytokine production [21]. In human infections such as *A. lumbricoides* and hookworms, which have some analogy to *N. brasiliensis* infections, the parasites are not confined to the intestine. Here larval migrations through the circulatory and pulmonary systems have resulted in these sites playing important roles in infection induced pathology and parasite killing [7]. Studies with *N. brasiliensis* show host responses in the lung play a key role in the rapid resolution of re-infection [7], [22]. Furthermore, roles for eosinophils [23], basophils [24] and CD4^+^ T cells [16], [25], but not B cells [20], in coordinating this immunity have also been demonstrated.

The work we present here addresses how B cells in secondary lymphoid organs (lymph nodes and spleen) can rapidly contribute to the control of recall immunity to *N. brasiliensis* re-infection. We then test if transfer of these potentially protective B cells can confer protection in a naïve mouse against primary exposure to *N. brasiliensis* infection. Using this approach, we identified key roles for B cells in immunity to the parasite. Strikingly, this was associated with production of IL-13 by IL-4Rα-sufficient B cells and also required MHCII compatibility and MyD88-expression. Thus, B cells can rapidly contribute to immunity to pathogens through multiple effector functions.

## Results

### IL-4Rα is essential for immunity against re-infection with *N. brasiliensis*

**Figure 1:**
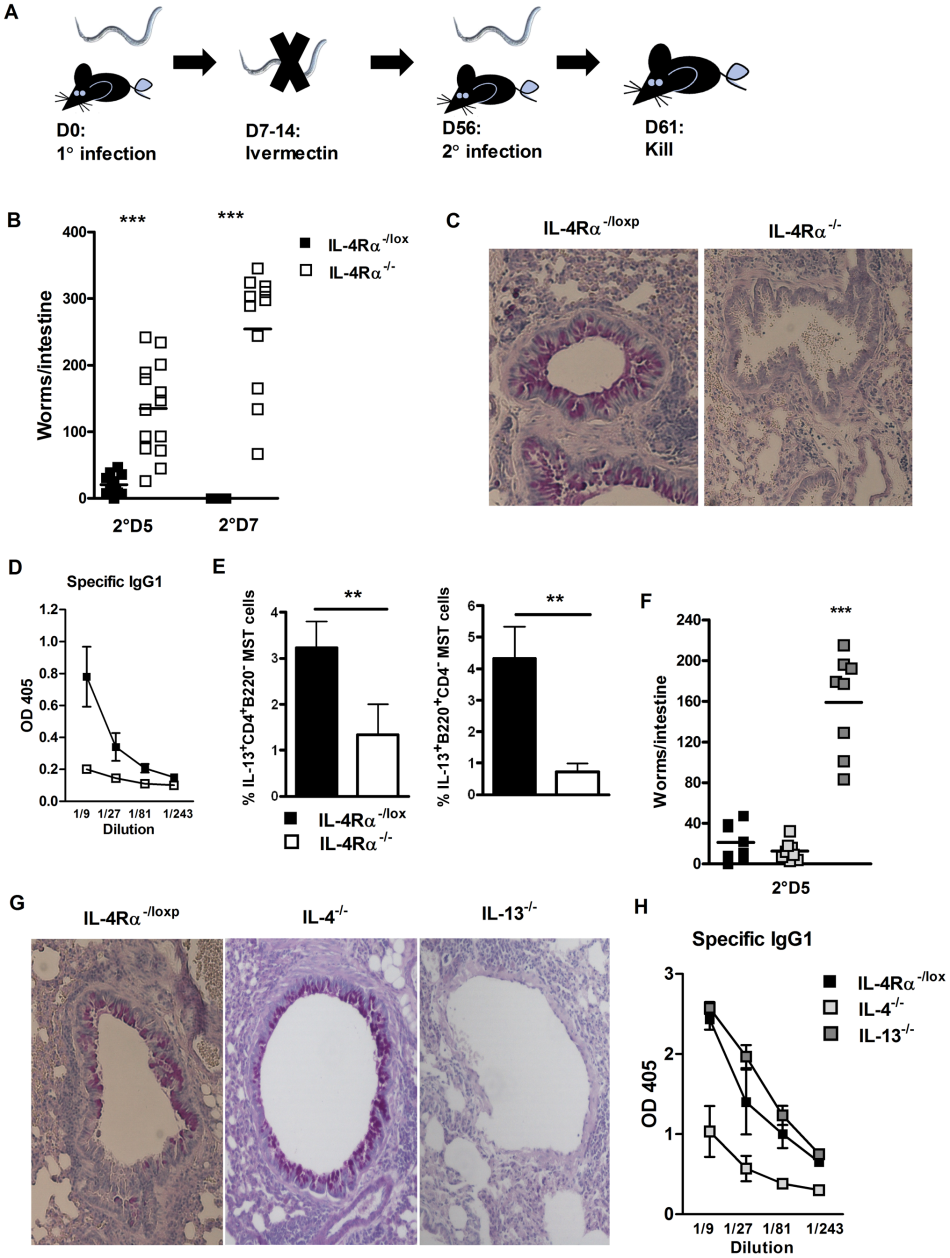
Protective immunity to *N. brasiliensis* re-infection is IL-13 and IL-4Rα dependent. IL-4Rα^−/−^ and IL-4Rα^−/lox^ mice were infected for 5 or 7 days post-secondary *N. brasiliensis* infection (**A**). Intestinal worm burdens were then quantified (**B**). Pulmonary mucus production was established by PAS staining (**C**). Serum Antibody titers of *N. brasiliensis* specific IgG1 were determined by ELISA (**D**). Mediastinal lymph node IL-13 responses were established by intracellular FACS staining in CD4^+^ T-cell and B220^+^ B-cell populations (**E**). IL-4^−/−^, IL-13^−/−^ and IL-4Rα^−/lox^ mice were infected for 5 days post-secondary *N. brasiliensis* infection and intestinal worm burdens were then quantified (**F**). Pulmonary mucus production was established by PAS staining (**G**). Serum Antibody titers of *N. brasiliensis* specific IgG1 were determined by ELISA (**H**). Data is representative of 3–4 independent experiments. n = 4–6 mice per group.

To identify a possible role for IL-4Rα in generating protective immunity IL-4Rα^−/lox^ (IL-4Rα sufficient) and IL-4Rα^−/−^ mice were infected with *N. brasiliensis*. Infection was subsequently cleared by drug treatment before re-infection with 500 L3 larva. Intestinal worm burden was quantified at different time points post-secondary infection (**Figure 1A**). IL-4Rα^−/−^ mice had significantly higher intestinal worm burdens compared to IL-4Rα^−/lox^ mice at day 5 or 7 post-secondary infection (**Figure 1B**). Loss of IL-4Rα was associated with multiple defects in known effectors of host T_H_2 immunity, including decreased mucus production in the lung (**Supplementary Figure S1A**), decreased IgG1 production and decreased IL-13 production by CD4 T cells and B cells in the lung draining lymph node (**Figure 1C–E**).

### IL-4Rα dependent immunity to *N. brasiliensis* re-infection is driven by IL-13 signalling

To investigate whether resolution of secondary *N. brasiliensis* infection was dependent on IL-4 and/or IL-13 signalling via IL-4Rα re-infection studies were repeated in IL-4^−/−^ and IL-13^−/−^ mice. Here, the significantly higher intestinal worm burden at day 5 secondary infection in IL-13^−/−^ mice compared to IL-4^−/−^ mice (**Figure 1F**), demonstrated IL-13 signalling through IL-4Rα is essential for immunity against re-infection with *N. brasiliensis*. This higher worm burden could was also be associated with an absence in goblet cell mucus production in IL-13^−/−^ mice, but not IL-4^−/−^ mice (**Figure 1G** **and Figure S1B**). IL-13^−/−^ mice demonstrated equivalent *N. brasiliensis* specific IgG1 antibody titers as IL-4Rα^−/lox^ mice. Conversely, IL-4^−/−^ mice demonstrated reduced specific IgG1 responses (**Figure 1H**). Taken together, these data (**Figures 1**) indicate that IL-4-depedent antigen specific IgG1 antibody responses may not be required for optimal immunity to re-infection, but that IL-4Rα-mediated IL-13 signalling is required.

### IL-4Rα-responsive B cells producing IL-13 are required for effective immunity to *N. brasiliensis* re-infection

**Figure 2:**
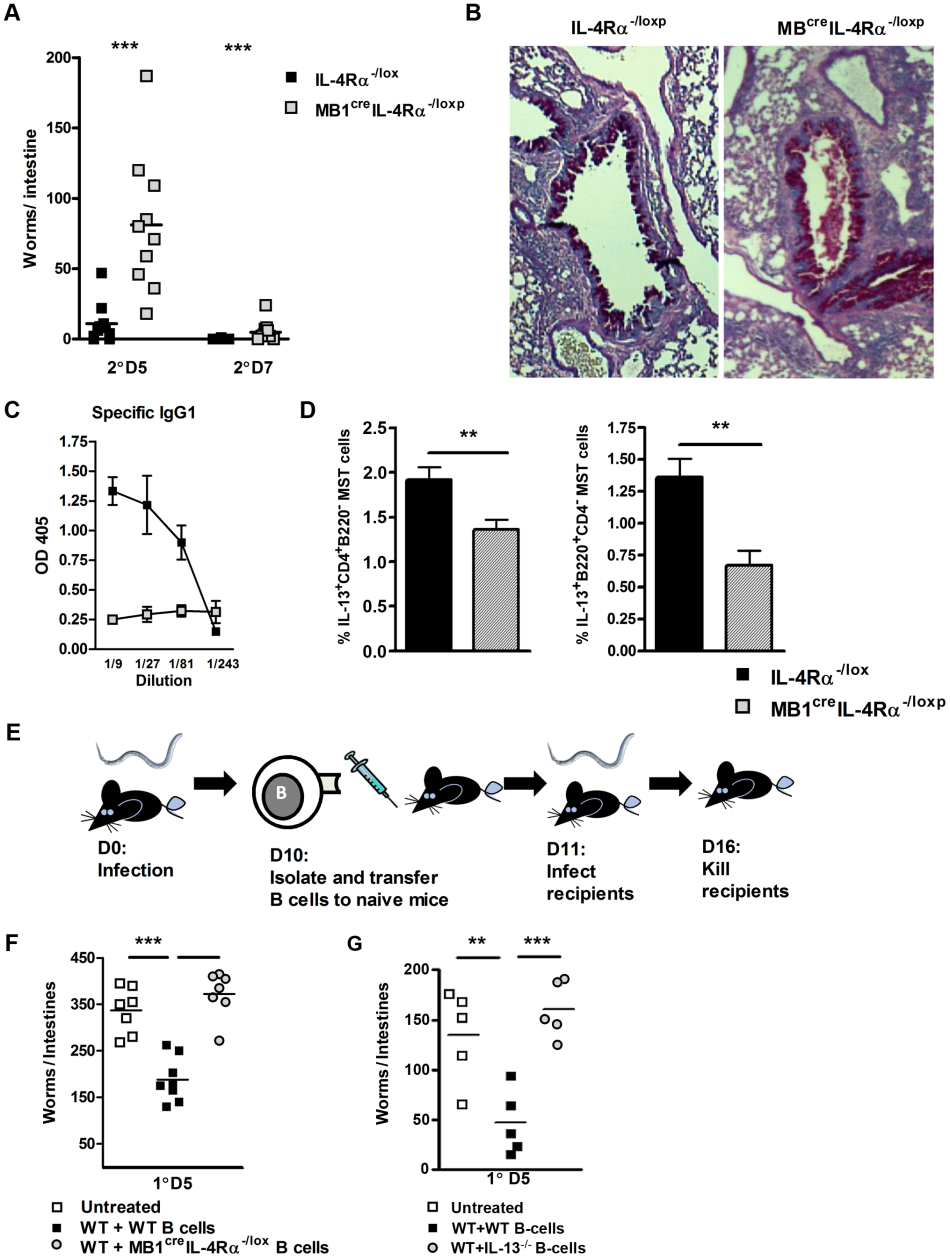
B cell IL-4Rα expression is required for optimal immunity to *N. brasiliensis* re-infection. *MB*1^Cre^IL-4Rα^−/lox^ and IL-4Rα^−/lox^ mice were infected for 5 or 7 days post-secondary *N. brasiliensis* infection and intestinal worm burdens were then quantified (**A**). Pulmonary mucus production was established by PAS staining (**B**). Serum Antibody titers of *N. brasiliensis* IgG1 were determined by ELISA (**C**). Mediatstinal lymph node IL-13 responses were established by intracellular FACS staining in CD4^+^ T-cell and B220^+^ B cell populations (**D**). B cells were isolated from *N. brasiliensis* infected BALB/c, *MB* ^cre^IL-4Rα^−/lox^ and IL-13^−/−^ and transferred into naïve BALB/c mice (**E**). Mice were then infected with 500xL3 *N. brasiliensis* larvae and worm burdens were then established at day 5 post infection (**F & G**). The results shown represent 2–4 independent experiments. n = 4–7 mice per group.

IL-4Rα-mediated effects on B cell function during *N. brasiliensis* re-infection were investigated in *MB1* ^Cre^IL-4Rα^−/lox^ BALB/c mice, which have B cell-specific abrogation of IL-4Rα expression [26]. Secondary infection resulted in a significantly higher intestinal worm burden in *MB1* ^Cre^IL-4Rα^−/lox^ mice when compared to IL-4Rα^−/lox^ mice (**Figure 2A**). Whilst goblet cell hyperplasia in *MB1* ^Cre^IL-4Rα^−/lox^ mice was equivalent to that seen in control IL-4Rα^−/lox^ mice (**Figure 2B** **and Figure S1C**). Antigen specific IgG1 was significantly drastically reduced (**Figure 2C**). Interestingly, IL-13 cytokine production by T and B cells was also reduced in *MB1* ^Cre^IL-4Rα^−/lox^ mice, when compared to IL-4Rα^−/lox^ mice (**Figure 2D** **and Figure S2**). Together, these studies show that a loss of IL-4Rα on B cells is sufficient to impair immunity to *N. brasiliensis* re-infection.

To demonstrate if IL-4Rα responsive and IL-13 competent B cells can directly confer protection against primary *N. brasiliensis infection*, we adoptively transferred B cells isolated from infected IL-4Rα^−/lox^, IL-13^−/−^ or *MB1* ^Cre^IL-4Rα^−/lox^ mice into naïve BALB/c mice (**Figure 2E** **and Figure S3**). Transfer of antigen-experienced IL-4Rα-responsive B cells into naïve BALB/c mice (WT+WT B-cells) reduced intestinal worm burdens. In contrast, transfer of primed B cells deficient for either the IL-4Rα or IL-13 did not reduce intestinal worm burden (**Figure 2F, G**). These results support our previous observations (**Figures 1** **&** **2A**) that IL-4Rα-responsive and IL-13 competent B cells contribute to protective immunity against *N. brasiliensis*.

**Figure 3:**
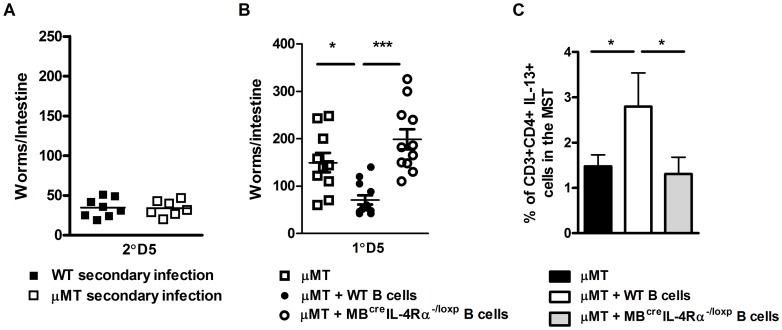
Transfer of *N. brasiliensis* experienced B cells enhances immunity to N. brasiliensis independently of endogenous B cell populations. *N. brasiliensis* infected μMT and BALB/c mice were re-infected with 500xL3 larvae and at day 5 post-secondary infection, the intestinal worm burdens was quantified (**A**). The possible role for IL-4Rα expressing B cells in boosting immunity independently of endogenous B cells was determined by transfer of B cells isolated from *N. brasiliensis* infected IL-4Rα^−/lox^ (WT B cells) or *MB*1^Cre^ IL-4Rα^−/lox^ (IL-4Rα^−/−^ B cells) into naïve μMT mice. These mice were then infected with 500xL3 *N. brasiliensis* and worm burdens quantified at day 5 post infection (**B**). Mediastinal lymph node CD3^+^CD4^+^ T cell populations IL-13 responses (**C**) were established by FACS staining. Results shown represent 2 independent experiments. n = 4–7 mice per group.

Previous studies have shown that in the absence of B cells control of *N. brasiliensis* re-infection is similar to WT mice [20]. Our experiments also found that the host's ability to control *N. brasiliensis* re-infection did not require B cells (**Figure 3A**). Nevertheless, this does not exclude the possibility that antigen-experienced B cells are able to modulate the response in normal mice. To test this experimentally, we transferred B cells from infected IL-4Rα^−/lox^ or infected *MB1* ^Cre^IL-4Rα^−/lox^ mice into naive μMT mice (**Figure 3B**). Transferred *N. brasiliensis* primed IL-4Rα responsive B cells augmented protection in μMT mice (**Figure 3B**), whereas transferred B cells from infected *MB1* ^Cre^IL-4Rα^−/lox^ mice did not. This B cell IL-4Rα-dependent protection correlated with significant increases in MST CD4^+^ T cell IL-13 production (**Figures 3C**). Together these results show that the immune response can compensate for the absence of B cells, but the introduction of pathogen-experienced IL-4Rα-responsive B cells can accelerate protective immunity against *N. brasiliensis*.

### Enhanced B cell ability to interact with T helper cells is required for optimal immunity to *N. brasiliensis* re-infection

**Figure 4:**
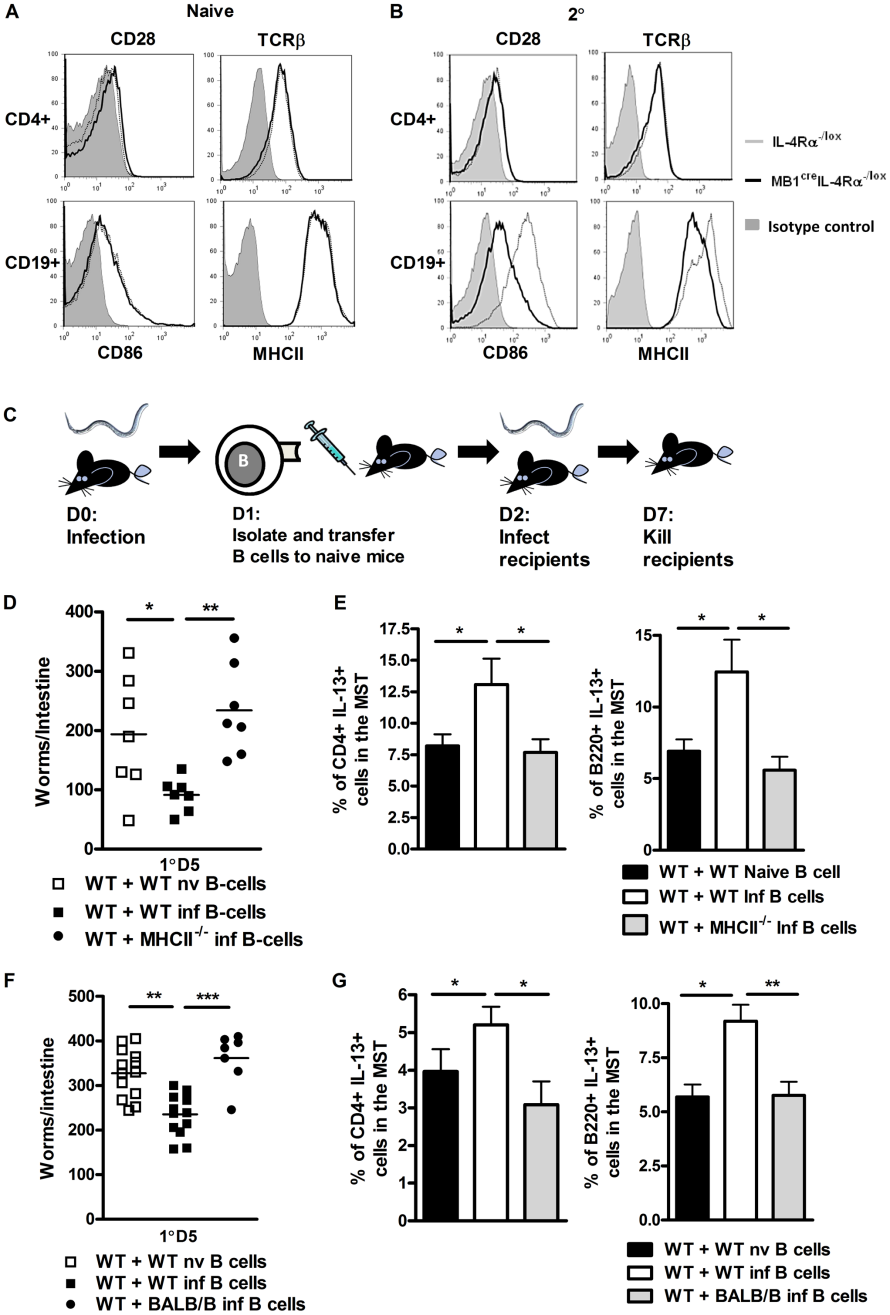
B cell MHCII antigen presentation mediates optimal immunity to *N. brasiliensis*. Surface expression of CD28 and TCR on CD4^+^ T cells and CD86 and MHCII on B cells in naive (**A**) and *N. brasiliensis* re-infected (**B**) IL-4Rα^−/lox^ mice and *MB1* ^Cre^IL-4Rα^−/lox^ mice was established by FACS analysis. Histograms: filled gray: isotype control, thin line: IL-4Rα^−/lox^, thick black line: *MB*1^Cre^IL-4Rα^−/lox^. Contributions by MHCII dependent antigen presentation were demonstrated by isolating WT or MHCII^−/−^ B cells from naive or infected mice then adoptively transferring into naive C57BL/6 mice (**C**). Mice were then infected with 500xL3 *N. brasiliensis* larvae and worm burdens were established at day 5 post infection (**D**). Mediastinal lymph node IL-13 responses were established by intracellular FACS staining in CD4^+^ T-cell and B220^+^ B cell populations (**E**). MHC dependent antigen presentation was confirmed by isolating WT and BALB/b B cells from naive or infected mice adoptively transferring into naive BALB/c mice. Mice were then infected with 500xL3 *N. brasiliensis* larvae and worm burdens were established at day 5 post infection (**F**). Mediastinal lymph node IL-13 responses were established by intracellular FACS staining in CD4^+^ T-cell and B220^+^ B cell populations (**G**). Data is representative of 2 independent experiments. n = 4–6 mice per group.

Optimal host control of *N. brasiliensis* re-infection was associated with B cell IL-13 production and enhanced CD4^+^ T cell T_H_2 responses. A further way B cells could contribute to T cell responses is through cognate, physical interactions that are associated with antigen-presentation through MHCII and co-stimulatory molecule expression. As a first step in demonstrating, if such an interaction is also a feature of IL-4Rα-dependent immunity to *N. brasiliensis* re-infection, we initially assessed expression of CD86 and MHCII on CD19^+^ B cells from naive and *N. brasiliensis* re-infected mice. CD86 and MHCII expression was equivalent between naive mice of both groups (**Figure 4A** **, lower panel**). In re-infected mice, CD86 and MHCII surface expression in *MB1* ^Cre^IL-4Rα^−/lox^ mice was reduced when compared with IL-4Rα^−/lox^ mice (**Figure 4B** **, lower panel**). CD4^+^ T cells showed no differences between the mouse strains in expression of CD28 and TCR. (**Figure 4A and B** **, upper panel**). Thus, IL-4Rα expression in B cells can help enhance the expression of B cell markers of activation after *N. brasiliensis* infection.

These findings indicated B cell cognate interactions with T cells and also antigen presentation may contribute to optimal immunity against *N. brasiliensis* re-infection. Our data presented in Figures 1 and 2 also indicates that B cell immunity may be independent of antibody class switching. This may exclude involvement of highly specific clonally expanded populations of B cells. We therefore hypothesized that protection may instead be mediated by a rapidly modified B cell antigen presenting response to *N. brasiliensis* infection. To demonstrate the possible role for antigen presentation, we adoptively transferred MHCII^−/−^ B cells from 1 day *N. brasiliensis* infected mice (**Figure 4C**) into naive mice. At the next day, mice were infected and days 5 post infection, recipients of MHCII^−/−^ B cells showed significantly higher worm burdens than mice, which received control WT B cells (**Figure 4D**). Protection was associated with increased IL-13 production by both B and T cell populations in the mediastinal lymph node (**Figure 4E**). To further control MHCII dependency, similar infection experiments were carried out in BALB/b mice, which are unable to present antigen via MHCII to BALB/c B cells. BALB/c recipients of adoptively transferred *N. brasiliensis*-experienced BALB/b B cells showed also significantly higher worm burdens, when compared to mice which received BALB/c B cells from *N. brasiliensis* infected mice (**Figure 4F**). These results further support that MHCII-dependent antigen presentation by B cells does contribute to host immunity to *N. brasiliensis*. Again, protection was associated with increased IL-13 production by both B and T cell populations in the mediastinal lymph node (**Figure 4G**). Together, these results suggest that MHCII expression contributes to the B cell protective response to *N. brasiliensis* re-infection.

### B cells can rapidly launch protective antigen-dependent responses to *N. brasiliensis* infection

**Figure 5:**
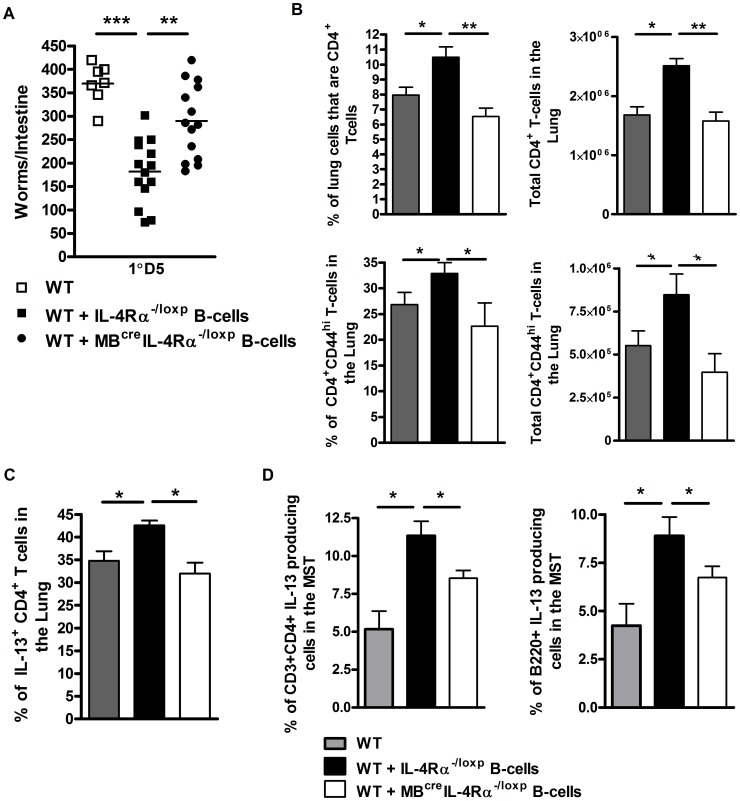
Rapid IL-Rα dependent B cell mediated protection against *N. brasiliensis* occurs in the lung. MB^cre^IL-4Rα^−/lox^ and IL-4Rα^−/lox^ mice were infected for 1 day with N. brasiliensis before spleen B cells were isolated and transferred into naive wild type mice (**As in** **Figure 4C**). These were infected with 500xL3 N. brasiliensis and intestinal worm burdens were quantified at day 5 post infection (**A**). Lung CD3^+^CD4^+^ and CD3^+^CD4^+^CD44^hi^ T cell populations were analysed by FACS staining (**B**). Lung CD4^+^ T cell (**C**) and mediastinal lymph node CD4^+^ T cell and B220^+^ B cell population (**D**) IL-13 responses were established by intracellular FACS staining. Data is representative of 2 independent experiments. n = 4–6 mice per group.

Our results presented in **Figure 4** suggested that antigen-experienced B cells can rapidly contribute to protection. We therefore tested *in vivo* whether immunity induced early in infection with *N. brasiliensis* is dependent on IL-4Rα-responsive B cells. Adoptive transfer of IL-4Rα responsive B cells from IL-4Rα^−/loxp^ mice isolated from mice 1 day post *N. brasiliensis* infection into wild type mice (**Figure 4C**) [27], [28] enhanced protection (**Figure 5A**), but not adoptive transfer of IL-4Rα unresponsive B cells from MB1^cre^IL-4Rα^−/loxp^ mice. Furthermore, only transfer of IL-4Rα responsive B cells enhanced B and CD4^+^ T cell IL-13 responses in the lung (**Figure 5B, C**) and mediastinal lymph node (**Figure 5D**), strengthen the necessity of IL-4Rα-responsive B cells for protective immunity.

This ability of B cells to confer protection so rapidly after parasite exposure further supports this response being independent of BCR. Other mechanisms of more rapid and possibly less stringent/polyfunctional antigen recognition by B cells may therefore play a role. Initial analysis does not support these transferred B cells conferring protection via an early production of IL-13 (**Figure S5**). However, rapid antigen processing and presentation may be mediated by B cells directly loading soluble peptide onto MHCII [29], [30] or via antigen internalisation and processing by Toll like receptors (TLR) [31], [32], [33].

**Figure 6:**
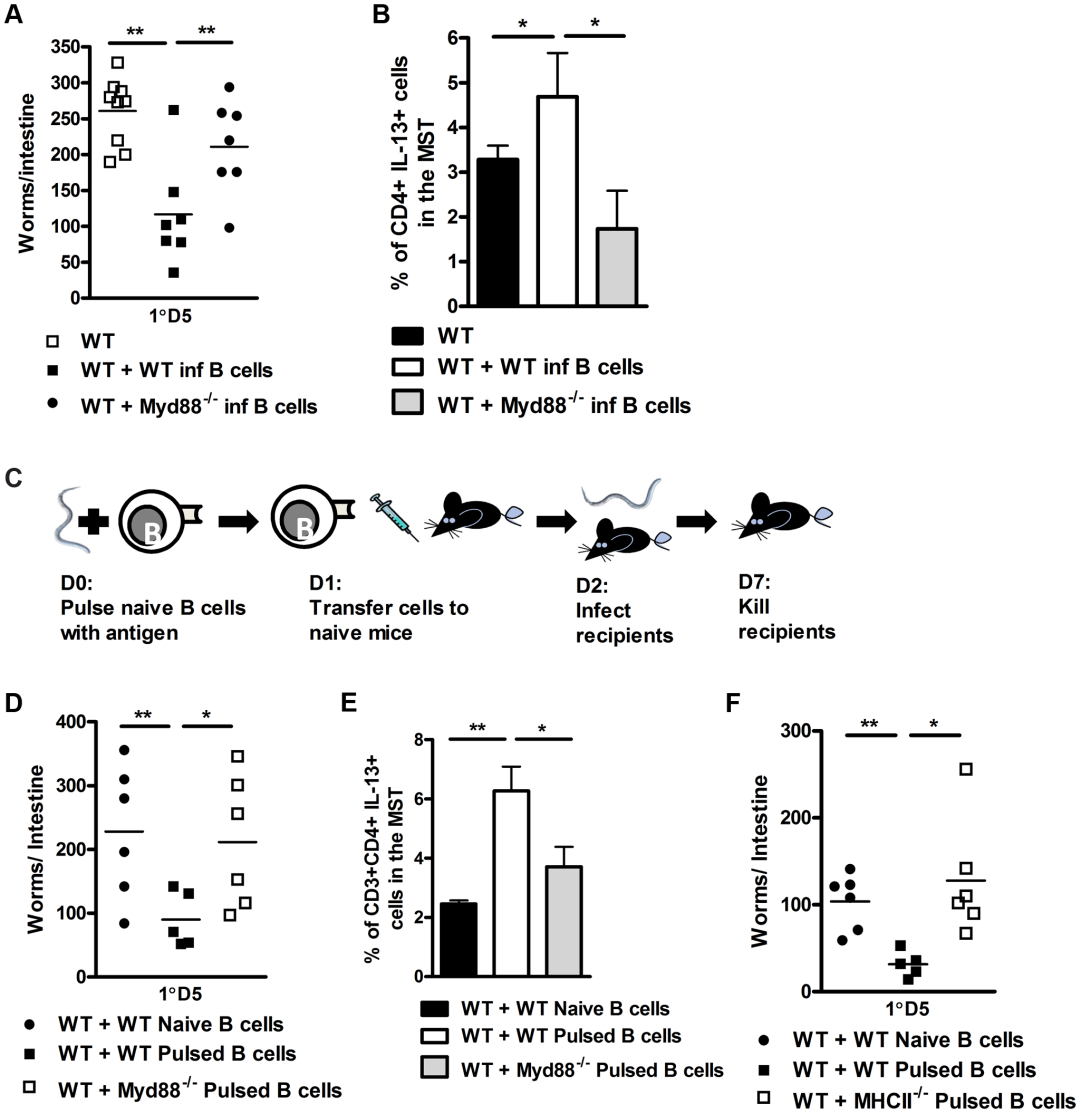
B cell MyD88 expression dependent protection against *N. brasiliensis* infection. B cells were isolated from WT or MyD88^−/−^ mice 24 hours post *N. brasiliensis* infection and adoptively transferred into naive WT mice (**As in** **Figure 4C**). 24 hours later these mice were infected with 500xL3 *N. brasiliensis* and subsequently killed 5 days post infection and worm burdens were counted (**A**). The mediastinal lymph node CD4^+^ T-cell IL-13 response was established (**B**). B cells were isolated from naive C57/BL6, MyD88^−/−^ or MHCII^−/−^ mice and pulsed with *N. brasiliensis* antigen overnight. These were then washed and transferred into naive C57/BL6 mice 24 h prior to infection (**C**). D5 PI intestinal worm counts are shown (**D & F**). The mediastinal lymph node CD4^+^ T-cell IL-13 response was established (**E**). Data is representative of 2 experiments, n = 5–7 mice per group.

To assess if rapid TLR mediated antigen processing contributed to reduced worm burdens, we repeated transfer experiments using B cells isolated from MyD88^−/−^ mice at one day post infection. Mice, which received MyD88^−/−^ B cells displayed significantly higher worm burdens than those which received B cells from WT 1 day infected recipients (**Figure 6A**). Protection was associated with increased IL-13 production by T cell populations in the mediastinal lymph node (**Figure 6B**). We then examined whether these effects were due to direct exposure of antigen by B cells by pulsing naive WT or MyD88^−/−^ B cells overnight with *N. brasiliensis* antigen (**Figure 6C**). Transfer of B cells from MyD88^−/−^ mice into naive mice resulted in impaired control of infection (**Figure 6D**), associated with lower IL-13 production by T cells in the mediastinal lymph node (**Figure 6E**). To rule out non-specific effects, we also pulsed wild type B cells with a range of antigens. We found that only B cells pulsed with *N. brasiliensis* antigen conferred a reduction in host intestinal worm burdens. Recipients of B cells pulsed with LPS (a potential bacterial contaminant during *N. brasiliensis* infection), ovalbumin and soluble *Leishmania major* antigen did not show any reduction in intestinal worm burden when compared to wild type controls (**Figure S6**). This data indicated that the reduction in worm burden that we see in recipients of *N. brasiliensis* pulsed B cells is pathogen specific. Moreover, pulsing of MHCII^−/−^ B cells with *N. brasiliensis* also resulted in impaired reduction in worm burdens (**Figure 6F**). Together these data suggest an association between rapid pathogen specific MyD88 dependent antigen processing and MHCII antigen presentation by B cells underlying the accelerated host immunity to *N. brasiliensis* infection.

## Discussion

This study demonstrated that IL-4Rα responsive B cells co-ordinate optimal immunity to secondary *N. brasiliensis* infection. This was related to B cell IL-13 expression, not IL-4 expression. B cell IL-4Rα mediated protection was associated with increased B cell and CD4 T cell IL-13 production. MHCII dependent B cell priming of T cells associated with this effect. Our data also demonstrated a rapid poly-functional antigen processing associated with B cell Myd88 expression.

B cell responses to *N. brasiliensis* have been suggested to be largely redundant [20]. Both our current study and that of Liu et al (18) demonstrate an absence of B cells *per se* does not alter host ability to control *N. brasiliensis* infection. However, we now show that a molecular change in B cell function, such as cell specific disruption of IL-4Rα expression on B cells, significantly impairs host ability to resolve *N. brasiliensis* infection. These findings also demonstrate important differences between B cell dependent immunity to *N. brasiliensis* and *H. polygyrus*. B cell Be2 immunity to *H. polygyrus* is dependent on B cell IL-4 production, B cell IL-4Rα expression and antigen presentation. As with *H. polygyrus*, *N. brasiliensis* re-infection is also dependent on B cell IL-4Rα expression and antigen presentation, however, B cell IL-13 production appears to play a functional role and not IL-4.

The protective B cell response we demonstrate may be independent of antibody and instead mediated through a B effector response. Such responses are particularly important in controlling CD4^+^ T cell driven immunity [34] via direct B and T cell interactions [35] as well as B cell cytokine production [36]. These B effector responses have an equivalent diversity in immune polarisation as T cells; producing Be1 (T_H_1) [35], Be2 (T_H_2) [36] and B_reg_ (T_reg_) [37], [38] effector B cells respectively. Functionally Be1 cells contribute significantly to immunity to bacterial infections, such as Salmonella [39], [40], [41]. In helminth infections Be2 cells have been demonstrated to be important for immunity to *H. polygyrus* [21], although humoral contributions also play a significant role [19], [20]. Evidence of B_regs_ induced by both *Heligmosomoides polygyus* and *Schistosoma mansoni* infection have elegantly shown helminth elicited B cell control of allergy [38], [42].

In this study we show that B cells develop a rapid and potent protective response against *N. brasiliensis* infection. This rapid protection precludes BCR-dependent clonal expansion following antigen exposure. Instead, it appears B cells are capable of responding to antigen via less stringent mechanisms than the BCR, such as direct peptide loading and Toll like receptors [29], [30]. Antigen binding by TLRs is established as an important regulator of B cell function [32]. TLR-dependent B cell responses can increase BCR-dependent antigen presentation [31], B cell cytokine production [39], [40] and play pivotal roles in B cell ability to interact with T cells [33]. These TLR mediated responses to antigen by B cells can be rapid and may not require clonal expansion of B cells [27].

In addition to antigen presentation we also demonstrate IL-4Rα-dependent increases in IL-13 production by endogenous B cells to be associated with control of secondary infection. This along with B cell-dependent induction of IL-13 production by endogenous CD4^+^ T cell and B cells would provide an important source of IL-13 to activate potential effector cell populations, including epithelial [13], [43], smooth muscle [12], [44] and innate immune cells [24], [45], [46].

In summary this study demonstrates IL-4Rα-responsive B cells playing an important role resolving secondary *N. brasiliensis* infection. We suggest the protective role played by B cells develops from antigen encounter with TLR driving an increase in CD86 and MHCII dependent interactions with CD4 T cells. This drives increased IL-13 production by CD4 T cells and B cells, facilitating host launching of protective mechanisms against *N. brasiliensis* infection.

## Methods

### Animals used

In this study the following BALB/c background mice were used: BALB/c, BALB/b, IL-4Rα^−/−^ [described as *Il4ra^tm1Fbb^/Il4ra^tm1Fbb^*], IL-13^−/−^, IL-4^−/−^and μMT. BALB/c background B cell specific IL-4Rα deficient MB1^Cre^IL-4Rα^−/lox^ [described as *Il4ra^tm1Fbb^/Il4ra^tm2Fbb^Tg (Cd79a^tm1(cre)Reth^)*] were generated as previously described [26]. MHCII^−/−^, MyD88^−/−^ and C57BL/6 mice were on C57BL/6 genetic background. Mice were bred and housed in specific pathogen–free conditions at the University of Cape Town, South Africa, and used in accordance with University Ethical Committee guidelines. All experimental mice were sex matched and used between 6–12 weeks of age with appropriate littermate controls of the same generation.

### Ethics statement

All studies were carried out under protocol 008/019 approved by the University of Cape Town Faculty of Health Sciences Animal Ethics Committee in accordance national guidelines laid down by the South African Board of Standards.

### *N. brasiliensis* infection

Mice were initially inoculated subcutaneously with 500 *N. brasiliensis* L3 larvae. At day 7 post infection worms were cleared by treatment with 10 mg/ml Ivermectin in drinking water for 7 days. Mice were then shelved for 21 days prior to a secondary subcutaneous infection with 500 *N. brasiliensis* L3 larvae. Mice were killed at day 5 post-secondary infection by CO_2_ inhalation. Adult worm burdens were determined as previously described [12]. Briefly, intestines were removed from infected mice and the lumen exposed by dissection. Intestines were then incubated at 37°C for 4 h in 0.65% NaCl. Intestinal tissue was then removed and the worms in the remaining saline solution counted.

### Histology

Tissue samples were fixed in a neutral buffered formalin solution. Following embedding in paraffin, samples were cut into 5 µm sections. Sections were stained with periodic acid-Schiff reagent (PAS) in order to visualise goblet cell hyperplasia [15]. The Histological Mucus Index (HMI) was used to quantify PAS positive airway epithelial cells. Sections photographed at 100× were overlaid with a standard grid. The number of grid units containing PAS positive epithelial goblet cells were divided by all units containing epithelial cells to establish the HMI.

### Determination of antibody titres

Parasite specific serum antibody levels from infected animals were determined as previously described [15]. Briefly, flat-bottom 96-well plates were coated overnight with 10 µg/ml of *N. brasiliensis* antigen. The plates were then washed and incubated in PBS containing 2% milk powder v/v for 1 h at 37°C. Following this, the plates were washed, samples loaded and incubated overnight at 4°C. Appropriate biotinylated secondary antibodies were then added following further washing and incubated overnight at 4°C. The plates were then washed, and antibody titres were determined using streptavidin-coupled horseradish peroxidase. The plates were developed with the TMB microwell peroxidase substrate system, and the reaction was stopped with 1 M H_3_PO_4_. The absorbance at 450 nm was determined with a Versamax microplate spectrophotometer (Molecular Devices, Germany).

### FACS analysis

The expression of surface receptors involved in B and T cell interactions were measured on mediastinal lymph node cells. Essentially, CD40-PE (clone 3/23), CD28-PE (clone 37.51) and TCRβ-biotin (clone H57-597) antibodies were used to detect receptors on CD4^+^ (clone GK1.5) T cells, while CD40L-PE (clone MR1), CD86-PE (clone GL1) and MHCII-bio (clone M5/114) were used to detect receptors on CD19^+^ (clone 1D3) B cells in IL-4Rα^−/lox^ and mb1^Cre^IL-4Rα^−/lox^ mice. Biotin-labeled antibodies were detected by streptavidin-APC, anti-FcR (clone 2.4G2) was used to block non-specific binding of immunoglobulins to the FcγII/III receptors and dead cells were excluded from analysis by 7-AAD staining (Sigma). Antibodies were from BD Pharmingen (San Diego, CA). Cells were acquired using a FACSCalibur (Beckton-Dickinson, Ferndale, South Africa) and data were analysed with Flowjo software (Treestar).

Intracellular cytokine staining was performed on mediastinal lymph node cells re-suspended in complete media (IMDM (GIBCO/Invitrogen; Carlsbad, CA), 10% FCS, P/S) at 2.5×10^7^/ml and stimulated with 10 µg/ml of *N. brasiliensis* antigen and GolgiStop (as per manufacturer's protocol; BD Pharmingen) at 37°C for 4 hours. After re-stimulation, cells were surface stained for CD3 (clone 500A2), CD4 (clone GK1.5) and B220 (clone RA3-6B2), then fixed and permeabilized with Cytofix/Cytoperm Plus (as per manufacturer's instructions; BD Pharmingen). Intracellular staining was performed by staining cells with IL-13-PE (ebio 13a) or appropriately labelled isotype control (eBioscience) [47].

### Sorting of B cells for adoptive transfer

Single cell suspensions from spleen were surface labelled with CD19 and B220 antibodies described above, re-suspended at 1×10^7^ cells/ml in media and sorted with a BD FACSARIA cell sorter (**Supplementary Figure S3A**). The purity of the isolated population was confirmed by flow cytometry, and samples showing <95% positive cells were discarded (**Supplementary Figure S3B**). Then the isolated B cells from naïve or infected mice were adoptively transferred to naive mice. The cells were re-suspended at 2.5×10^6^ cells/ml in media. Each mouse received 0.5×10^6^ B cells injected intravenously into the tail vein 24 h prior to infection with *N. brasiliensis*.

### In vitro B cell antigen pulsing

Naïve B cells were isolated from a single splenocyte suspension by FACSARIA as described above. Purity was confirmed by flow cytometry, samples showing <95% B220 positive cells were discarded (**Supplementary Figure S3**). Cells were incubated (pulsed) with 10 µg/ml *N. brasiliensis* antigen, ovalbumin, LPS or soluble Leishmania antigen for 16 h at 37°C. Cells were then washed 3× in media by centrifugation and then re-suspended in media at 2.5×10^7^/ml. 0.5×10^6^ cells were then transferred intravenously into naïve mice 24 h prior to infection with *N. brasiliensis* [27], [28].

### Generating *N. brasiliensis* somatic antigen

L3 larvae were washed from filter paper into H_2_O/50 µg/ml Penicillin+Streptomycin and allowed to stand for an hour during which the larvae settle to the bottom of the container, after which the larvae washed once more in H_2_O/Pen./Strep and once in H_2_O. Then the larvae were concentrated into 2 ml of distilled H_2_O and snap frozen in liquid nitrogen. Following this the preparation was homogenized for 5 to 10 minutes before the whole solution is centrifuged at 10 000 rpm for 10 minutes. The supernatant contains the soluble fraction of the L3 larvae proteins which is measured and standardised using a BCA protein assay (Pierce; Chicago, IL). Antigen was added to the cells in solution at 10 µg/ml.

### Statistics

Values are expressed below as means ± standard deviations and significant differences were determined using either Mann-Whitney U test or ANOVA (GraphPad Prism4).

## Supporting Information

Figure S1 **Lung epithelial mucous production is reduced in IL-4Rα^−/−^ and IL-13^−/−^ mice but not in IL-4^−/−^ or MB1^cre^IL-4Rα^−/lox^ mice.** IL-4Rα^−/−^, IL-4^−/−^, IL-13^−/−^, MB1^cre^IL-4Rα^−/lox^ and IL-4Rα^−/lox^ mice were infected for 5 days post-secondary *N. brasiliensis* infection. Pulmonary mucus production was established by PAS staining (**Figure 1** **and** **2**). The Histological Mucus Index (HMI) [15] was used to quantify the numbers of PAS positive epithelial cells.
(TIF)

Figure S2 **Spleen B cell IL-13 production is reduced in MB1^cre^IL-4Rα^−/lox^ mice.** Naïve *MB*1^Cre^IL-4Rα^−/lox^ and IL-4Rα^−/lox^ mice were infected with *N. brasiliensis*. Splenic B cell IL-13 responses at day 10 post infection were established by intracellular FACS staining in B220^+^CD19^+^ populations.
(TIF)

Figure S3 **Gating strategy for isolation and establishing purity of B cells.** The purity of B cells was established by flow cytometry using the gating strategy shown (**A**). Purity was over 95% in all cases (**B**). Briefly, lymphocytes were identified according to forward scatter vs. side scatter profile. CD11c^neg^, CD3^neg^, CD4^neg^ and GR-1^neg^ and B220^+^CD19^+^ cells were then isolated and used for further analysis..
(TIF)

Figure S4 **Transfer of naïve B cells does not confer protection against** ***N. brasiliensis*** **infection.** B cells isolated from naïve BALB/c mice were pulsed with antigen and transferred into naïve BALB/c mice. Mice were then infected with 500xL3 *N. brasiliensis* larvae and worm burdens were then established at day 5PI.
(TIF)

Figure S5 **B cell IL-13 cytokine responses at 1 day post** ***N. brasiliensis*** **infection.** Mice were infected with 500xL3 *N. brasiliensis* larvae and killed 1 day post infection. Splenic B cell IL-13 production was established by flow cytometry.
(TIF)

Figure S6 **B cell mediated immunity to** ***N. brasiliensis*** **is antigen specific.** B cells isolated from naïve BALB/c mice were pulsed with antigen and transferred into naïve BALB/c mice. Mice were then infected with 500xL3 *N. brasiliensis* larvae and worm burdens were then established at day 5PI. Antigen specific protection by B cells was established by pulsing B cells with *N. brasiliensis*, *L. major* or Ova antigens or LPS then adoptively transferring into naive BALB/c mice.
(TIF)
