# Supplementary figures and images for: IL-4Rα-Associated Antigen Processing by B Cells Promotes Immunity in Nippostrongylus brasiliensis Infection
Source: PLoS Pathog. 2013 Oct 24;9(10):e1003662. doi: 10.1371/journal.ppat.1003662 (PMC3812011; doi:10.1371/journal.ppat.1003662)

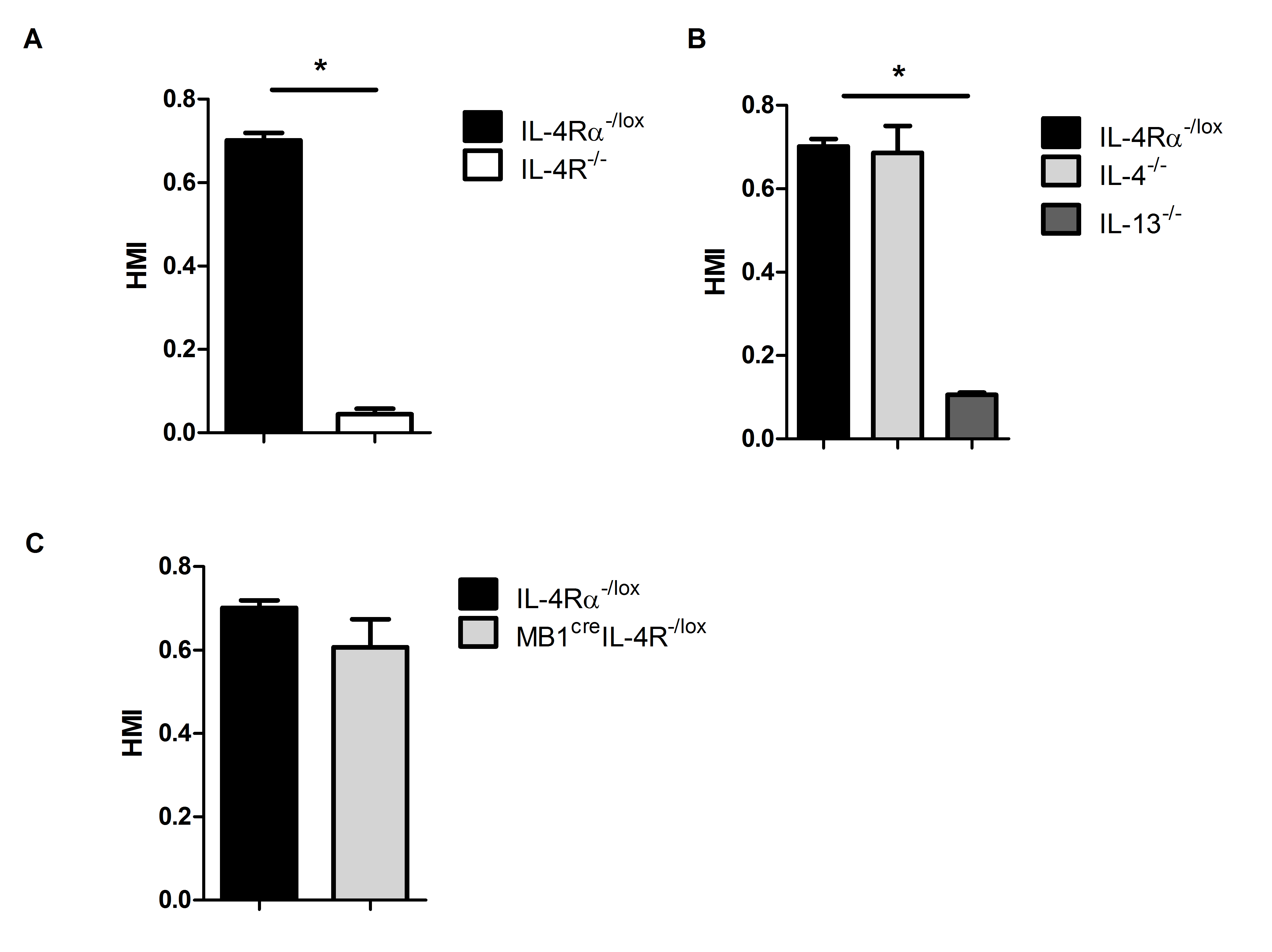

Supplement: Figure S1 — Lung epithelial mucous production is reduced in IL-4Rα−/− and IL-13−/− mice but not in IL-4−/− or MB1creIL-4Rα−/lox mice. IL-4Rα−/−, IL-4−/−, IL-13−/−, MB1creIL-4Rα−/lox and IL-4Rα−/lox mice were infected for 5 days post-secondary N. brasiliensis infection. Pulmonary mucus production was established by PAS staining ( Figure 1 and 2 ). The Histological Mucus Index (HMI) [15] was used to quantify the numbers of PAS positive epithelial cells. (TIF) [file ppat.1003662.s001.tif]

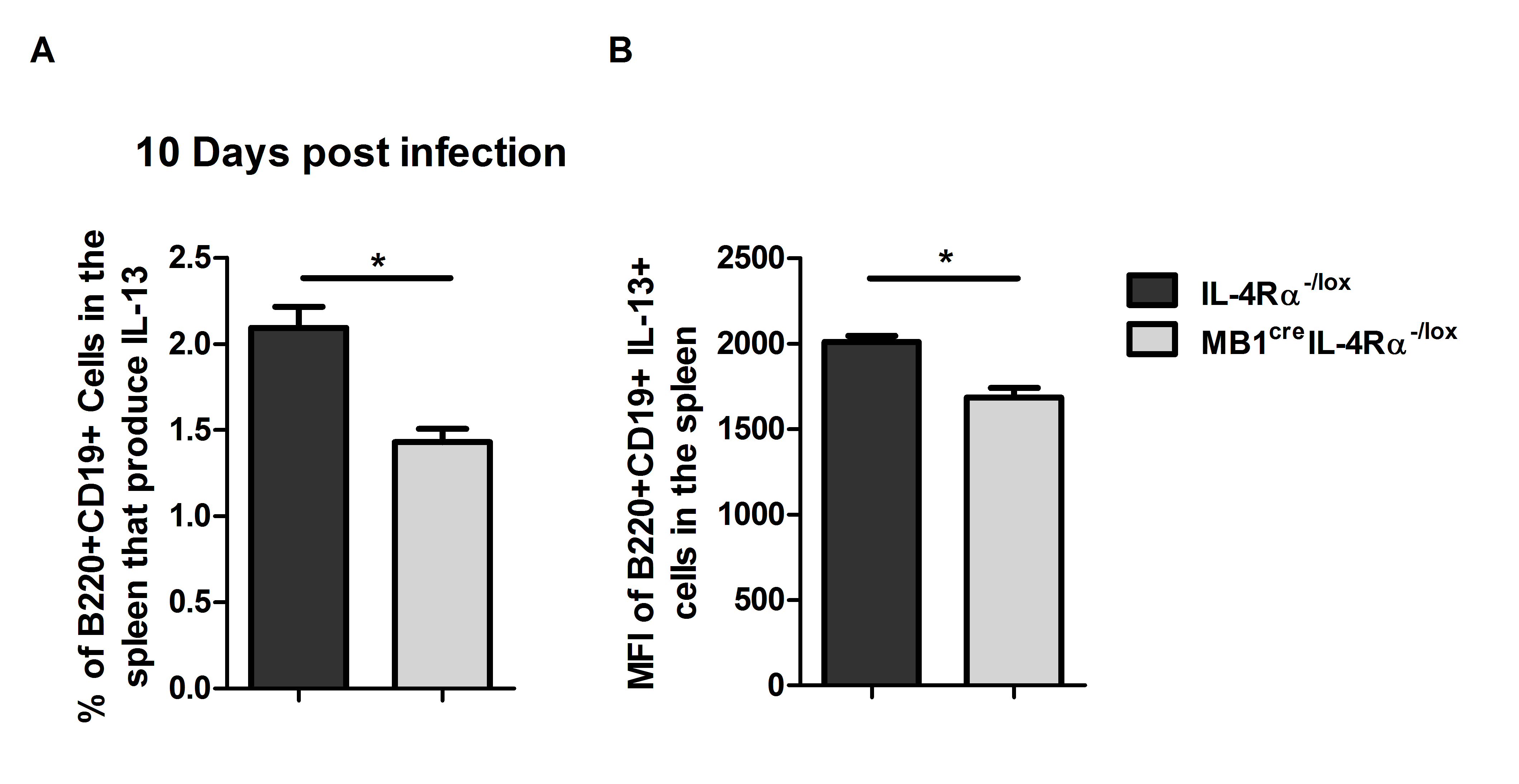

Supplement: Figure S2 — Spleen B cell IL-13 production is reduced in MB1creIL-4Rα−/lox mice. Naïve MB1CreIL-4Rα−/lox and IL-4Rα−/lox mice were infected with N. brasiliensis. Splenic B cell IL-13 responses at day 10 post infection were established by intracellular FACS staining in B220+CD19+ populations. (TIF) [file ppat.1003662.s002.tif]

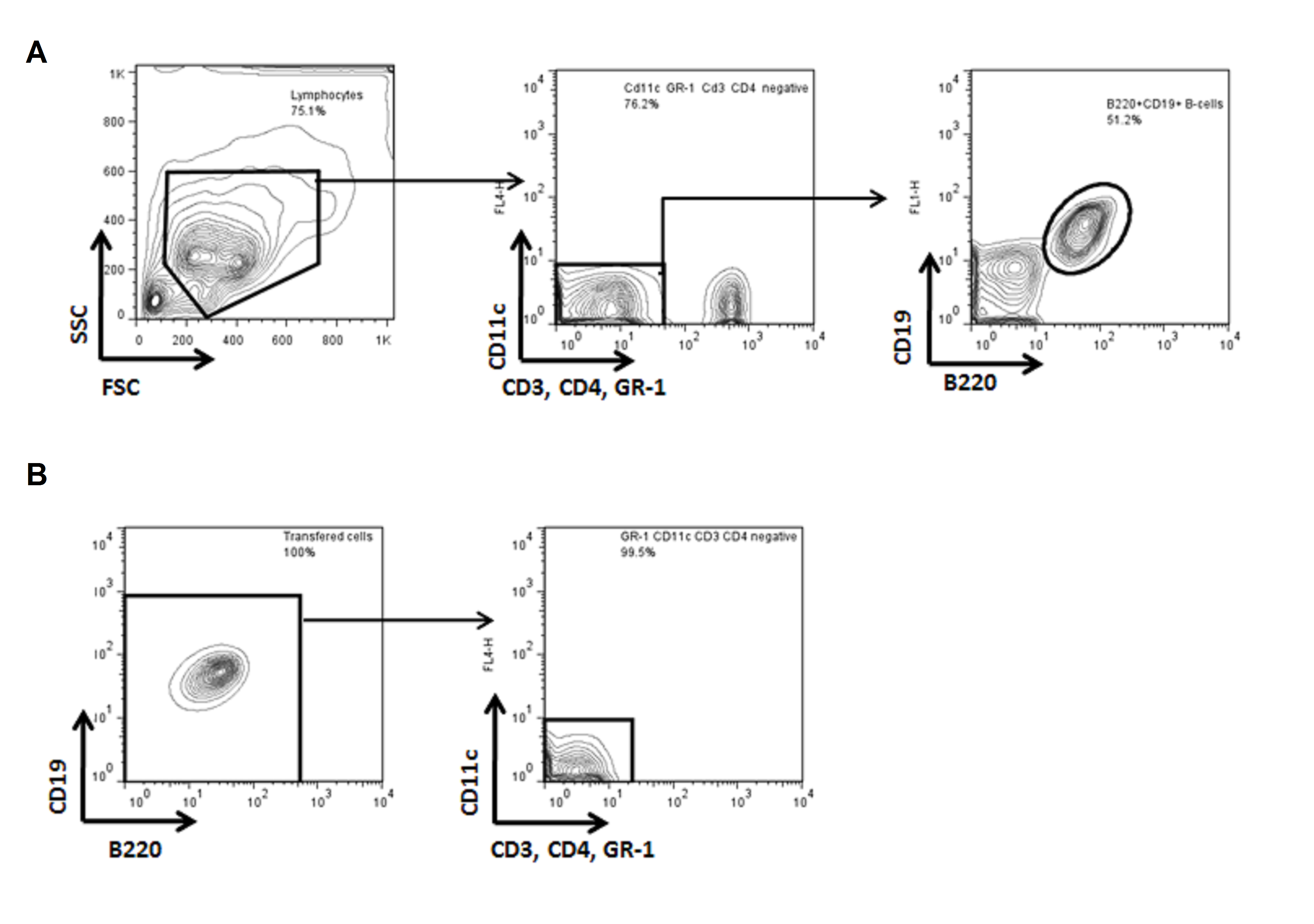

Supplement: Figure S3 — Gating strategy for isolation and establishing purity of B cells. The purity of B cells was established by flow cytometry using the gating strategy shown (A). Purity was over 95% in all cases (B). Briefly, lymphocytes were identified according to forward scatter vs. side scatter profile. CD11cneg, CD3neg, CD4neg and GR-1neg and B220+CD19+ cells were then isolated and used for further analysis.. (TIF) [file ppat.1003662.s003.tif]

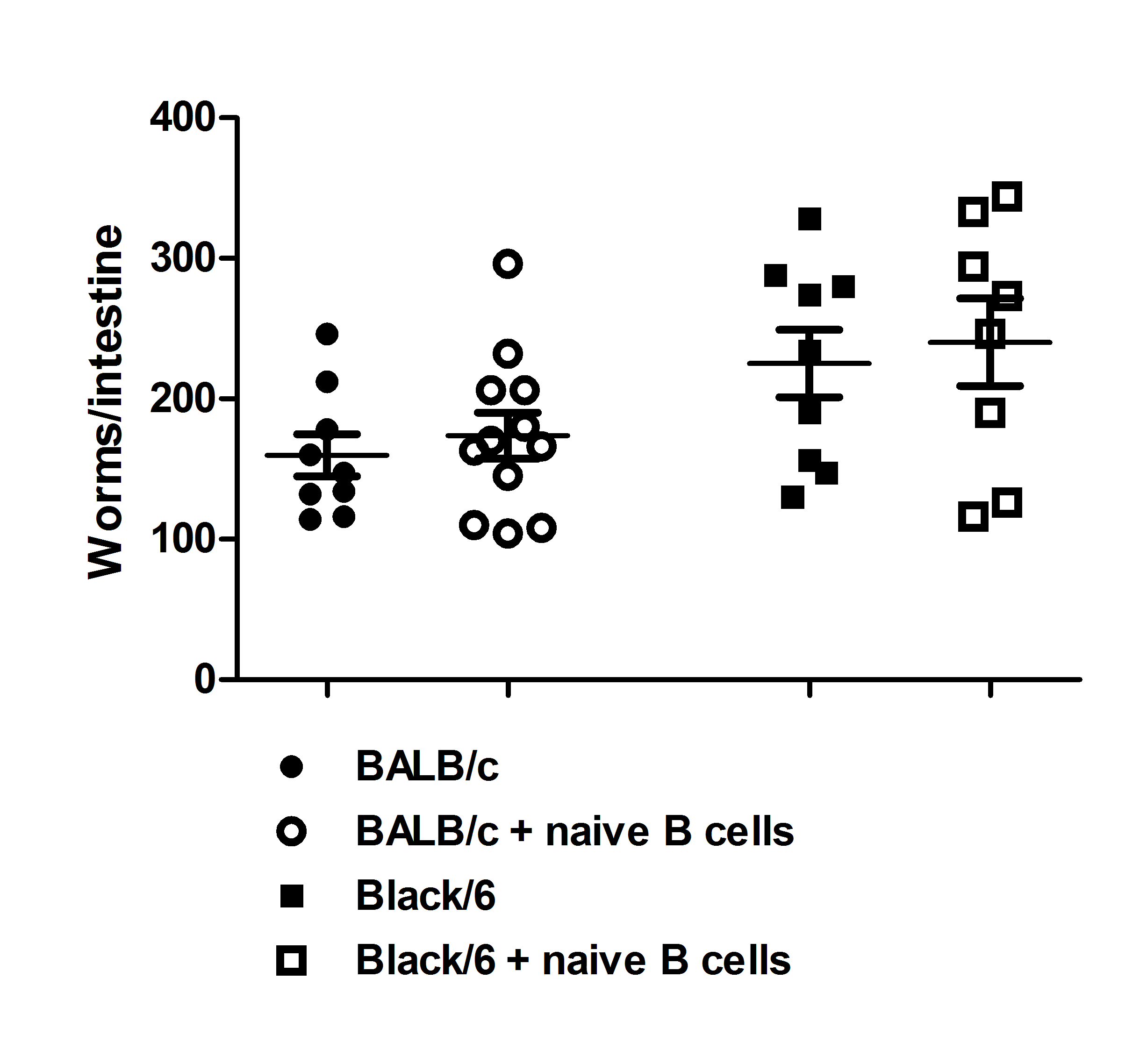

Supplement: Figure S4 — Transfer of naïve B cells does not confer protection against N. brasiliensis infection. B cells isolated from naïve BALB/c mice were pulsed with antigen and transferred into naïve BALB/c mice. Mice were then infected with 500xL3 N. brasiliensis larvae and worm burdens were then established at day 5PI. (TIF) [file ppat.1003662.s004.tif]

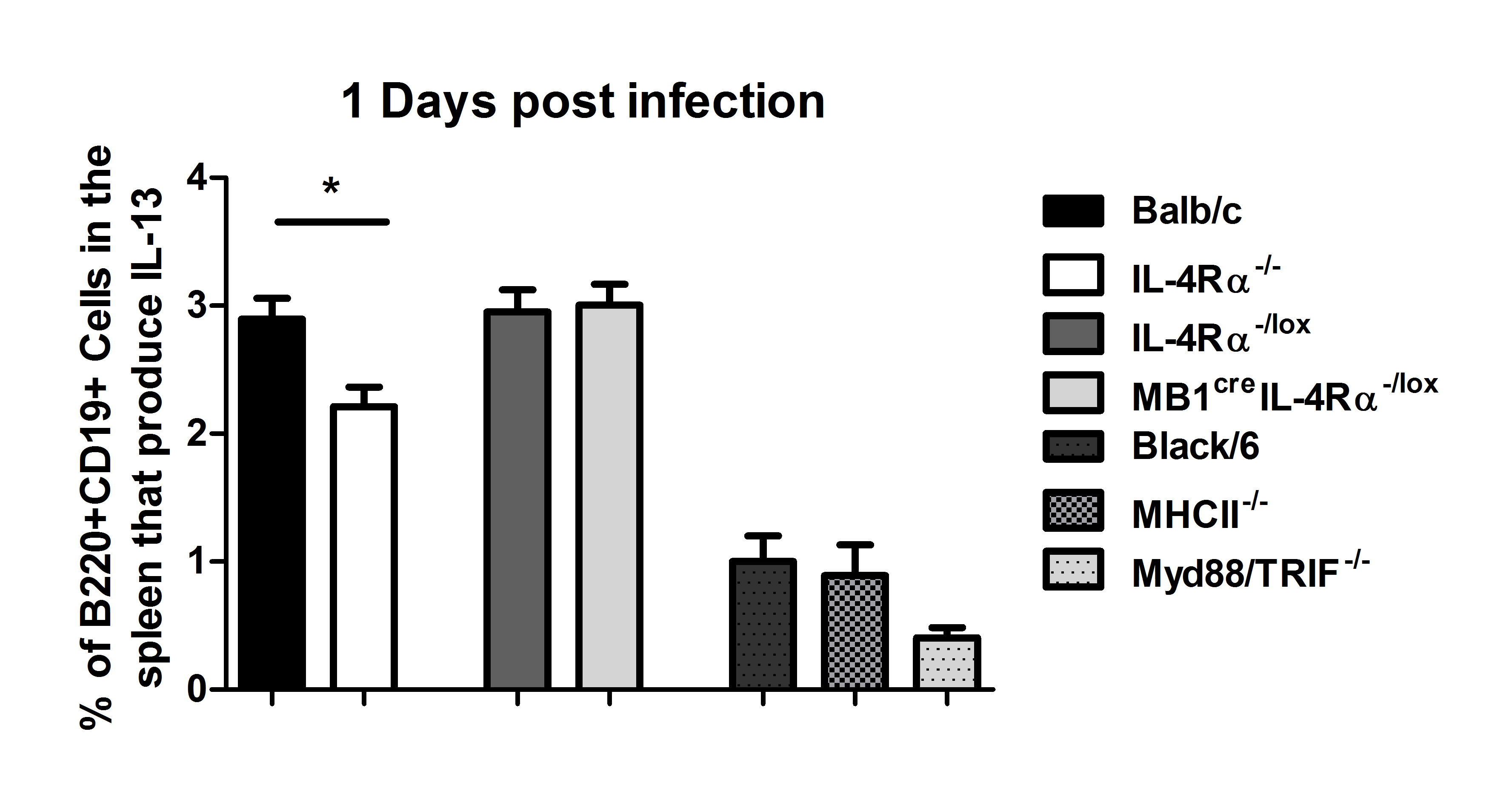

Supplement: Figure S5 — B cell IL-13 cytokine responses at 1 day post N. brasiliensis infection. Mice were infected with 500xL3 N. brasiliensis larvae and killed 1 day post infection. Splenic B cell IL-13 production was established by flow cytometry. (TIF) [file ppat.1003662.s005.tif]

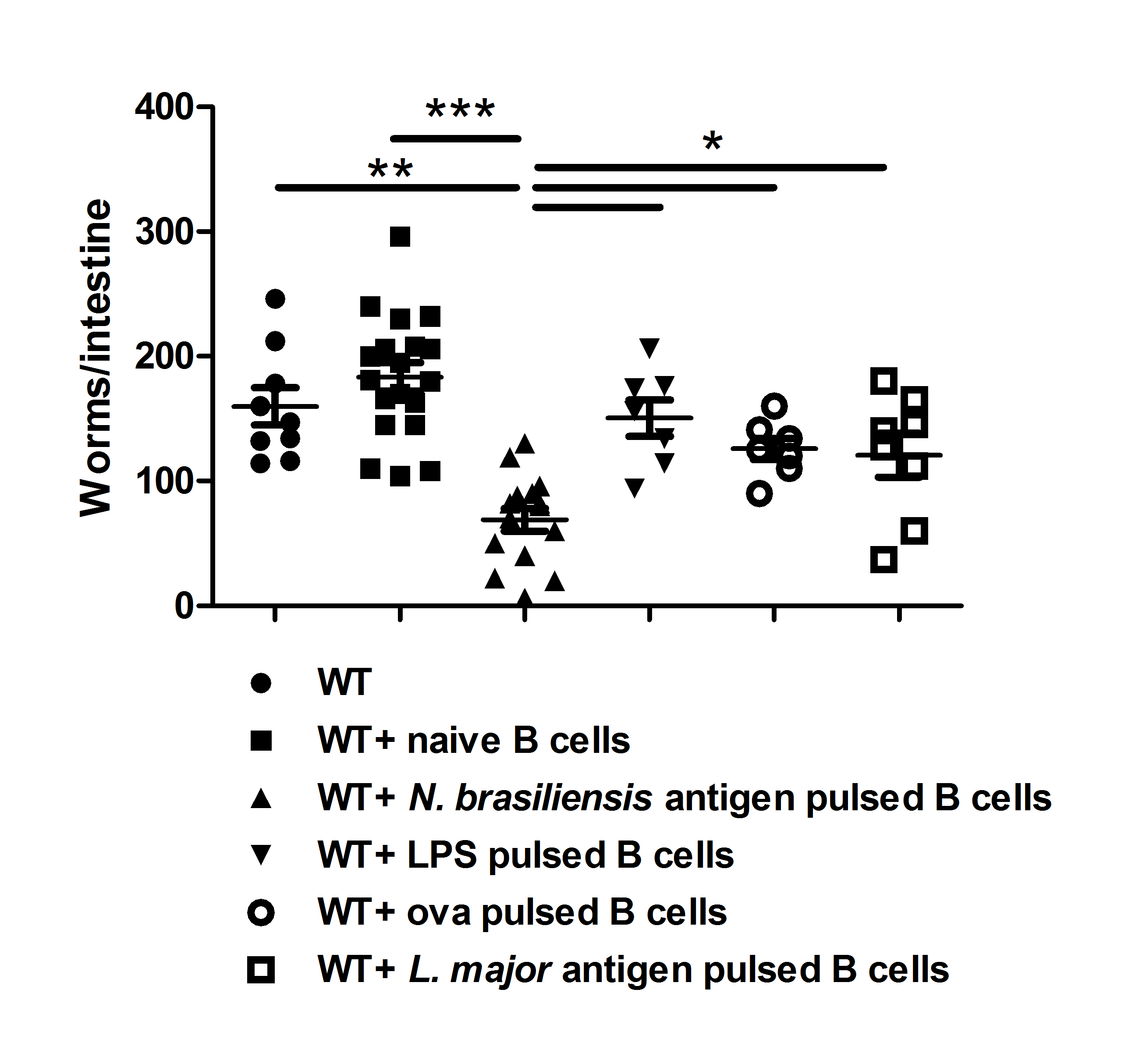

Supplement: Figure S6 — B cell mediated immunity to N. brasiliensis is antigen specific. B cells isolated from naïve BALB/c mice were pulsed with antigen and transferred into naïve BALB/c mice. Mice were then infected with 500xL3 N. brasiliensis larvae and worm burdens were then established at day 5PI. Antigen specific protection by B cells was established by pulsing B cells with N. brasiliensis, L. major or Ova antigens or LPS then adoptively transferring into naive BALB/c mice. (TIF) [file ppat.1003662.s006.tif]
